# Observational studies of depression in primary care: what do we know?

**DOI:** 10.1186/1471-2296-8-28

**Published:** 2007-05-11

**Authors:** Gail Gilchrist, Jane Gunn

**Affiliations:** 1 The Department of General Practice, The University of Melbourne, Carlton, Victoria, Australia

## Abstract

**Background:**

We undertook a systematic review of observational studies of depression in primary care to determine 1) the nature and scope of the published studies 2) the methodological quality of the studies; 3) the identified recovery and risk factors for persistent depression and 3) the treatment and health service use patterns among patients.

**Methods:**

Searches were conducted in MEDLINE, CINAHL and PsycINFO using combinations of topic and keywords, and Medical Subject Headings in MEDLINE, Headings in CINAHL and descriptors in PsycINFO. Searches were limited to adult populations and articles published in English during 1985–2006.

**Results:**

40 articles from 17 observational cohort studies were identified, most were undertaken in the US or Europe. Studies varied widely in aims and methods making it difficult to meaningfully compare the results. Methodological limitations were common including: selection bias of patients and physicians; small sample sizes (range 35–108 patients at baseline and 20–59 patients at follow-up); and short follow-up times limiting the extent to which these studies can be used to inform our understanding of recovery and relapse among primary care patients with depression. Risk factors for the persistence of depression identified in this review were: severity and chronicity of the depressive episode, the presence of suicidal thoughts, antidepressant use, poorer self-reported quality of life, lower self-reported social support, experiencing key life events, lower education level and unemployment.

**Conclusion:**

Despite the growing interest in depression being managed as a chronic illness, this review identified only 17 observational studies of depression in primary care, most of which have included small sample sizes and been relatively short-term. Future research should be large enough to investigate risk factors for chronicity and relapse, and should be conducted over a longer time frame.

## Background

A recent World Health Organization report states that depression is the leading cause of disability worldwide among people aged five and above [1]. People with depression are mainly managed in primary care/general practice [2], yet current management guidelines are mainly based upon data collected in the secondary and tertiary sectors. Studies of relapse rates, risk factors for relapse and efficacy of maintenance therapy have been conducted mainly in tertiary psychiatric settings [3], with a paucity of data from primary care [4,5].

In preparing for an observational study of the health service use and treatment patterns of Australians experiencing depressive symptoms [6] we undertook a systematic review of observational studies of depression in primary care to determine 1) the nature and scope of the published studies 2) the methodological quality of the studies; 3) the identified recovery and risk factors for persistent depression and 3) the treatment and health service use patterns among patients.

## Methods

### Selection and inclusion criteria

We selected prospective observational studies where primary care patients were screened for depression and followed over time. Articles were assessed as relevant by both authors. We identified three review articles that described the prevalence and course of depression in primary care [4,5,7], relevant studies from these reviews published during 1985–2006 are included in the current review.

### Exclusion criteria

As the purpose of this review was to examine naturalistic studies receiving routine care, subjects recruited as part of a randomised controlled trial were excluded to reduce bias from selecting patients consenting to participate in intervention or medication trials. It was considered that these patients may not be representative of patients in general practice, as research has found that many primary care patients prefer psychological treatments [8] and therefore may be less likely to enter antidepressant treatment or randomized clinical trials as a result [9]. It has also been shown that subjects in randomised trials have better care and outcomes than 'routine care'. Articles were excluded for the following reasons: patients were originally recruited as part of a randomised controlled trial (n = 11) [3,10-19] or an intervention study (n = 1) [20]; retrospective analyses of administrative or clinical data were used (n = 6) [21-26]; retrospective life charts were used to gain longitudinal data (n = 1) [27]; only primary care patients initiating antidepressant treatment were recruited (n = 2) [28,29]; patients selected were 'psychiatric cases' and results were not presented for depression (n = 2) [30,31]; and review articles (n = 1) [5].

### Search strategy

**Figure 1:**
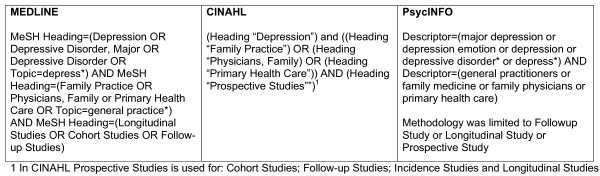
Search strategies limited to articles published in English during 1985–2006 and adult populations.

An Information Consultant at The University of Melbourne assisted with the development of the search strategy. Searches were conducted separately for each of the following databases: MEDLINE, CINAHL and PsycINFO. Search strategies were devised using combinations of topic and keywords, and Medical Subject Headings (MeSH) in MEDLINE, Headings in CINAHL and descriptors in PsycINFO. Searches were limited to adult populations and articles published in English during 1985–2006. Figure 1 describes the search statements conducted in each of the three databases searched. Further relevant articles were sourced through cross checking references in articles and from the authors' library.

## Results

**Figure 2:**
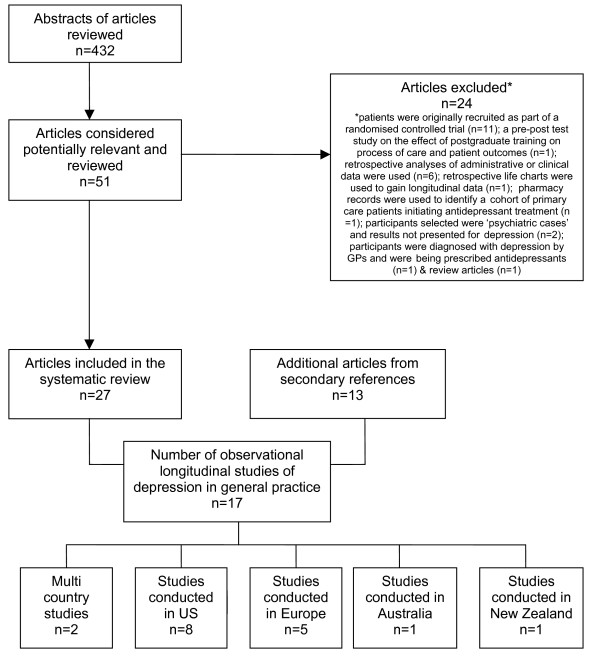
Flowchart of articles included in the review.

The abstracts of 432 articles identified using the search strategy described above were considered (Figure 2). Fifty-one potentially relevant articles were retrieved for comprehensive review. Twenty four articles were excluded as they did not meet criteria for an observational study of depression (see exclusion criteria). Forty articles from 17 observational prospective cohort studies were identified, 27 articles from the original search [32-58] and 13 from secondary references [59-71].

### Methods used

Table 1 describes the methods for the 17 longitudinal studies included in the review. The studies varied widely in their original purpose. The studies can be grouped into those that focused on depressive symptoms (n = 7) [45,51,54,61,63,66,68] and those that focused on major depressive disorder satisfying DSM IV criteria (n = 10) [37,38,43,44,48,49,59,64,67,69]. Nine of the 17 studies aimed to describe the course of depression over time and identify risk factors associated with recovery or improvement in depression [37,43,44,54,61,63,64,68,69]. Four studies were interested in examining detection of depression by the practitioner and depression outcome [38,49,64,66]. One study examined the seasonality prevalence and incidence of depressive disorder [59], one examined the process and outcomes of rural depression care [67], one examined the outcomes for cases 'missed' at the screening encounter [45], one examined the prevalence of bipolar II disorder with depressive and anxiety subtypes [48], and one examined whether managed care was associated with reduced access to mental health specialists and poorer outcomes among patients with depressive symptoms [51].

**Table 1:** Methods

**Author**	**Blacker et al. [59]**	**Grembowski et al. [51]**	**Groningen Primary Care Study [32-35,50,64,65]**	**Kessler et al. [61]**	**Kessler et al. [45,60]**	**Limosin et al. [43]**	**Longitudinal investigation of depression outcomes in primary care (LIDO) [39,40, 44,52,62]**	**Mental Health & General Practice Investigation (MaGPie) [63]**	**Manning et al. [48]**
**Location**	England	US	Netherlands	US	England	France	Israel, Brazil, Spain, Australia, Russia, US	New Zealand	US
**Primary care setting/s**	One health centre	261 primary physicians in private practice from 72 offices	25 General Practitioners	A multi-specialty group practice with 175 physicians	One general practice	560 General Practitioners	6 research sites Primary care & outpatient services, day care services, & inpatient hospital services	70 General Practitioners	One private ambulatory family practice centre
**Selection of primary care setting**	Not stated	Consenting GPs from Physician Referral Study	Representative sample	Not stated	Not stated	Randomly selected	Track record of international collaborative research	Randomly selected	Not stated
**Recruitment**	Consecutive patients	Waiting room	Consecutive patients	Patients who used clinic	Consecutive patients	Each GP enrolled first patient to meet criteria for major depressive episode	Patients attending primary care facilities	All adult attenders	Consecutive non-referred patients presenting with impairment due to depression or anxiety
**Screen for depression/mental health**	General Health Questionnaire (GHQ^)^-30, Schedule for Affective Disorders and Schizophrenia (SADS^)^	Symptom Checklist (SCL) -20	GHQ-30 & rated by GP for current mental health problem	GHQ-30	GHQ-12	Structured Clinical Interview for DSM (SCID)	Center for Epidemiologic Studies-Depression Scale (CES-D)	GHQ-12	5 question screening instrument
**Exclusion criteria**	Not stated	<18 years, non English speaking	Not stated	Not stated	Not stated	<18 years	Recent treatment for depression; psychoses; dementia; any other condition would interfere with the study objectives	< 18 years, not able to read English & consulting with GP other than index GP	Not stated
**Criteria for inclusion in cohort**	Depressive disorders	Depressive symptoms	Three or more psychiatric symptoms on PSE	192 patients with GHQ-30 scores = 4 & 55 with lower scores	Completion of GHQ	Major Depressive Episode & scored Montgomery-Åsberg Depression Rating Scale (MADRS) ≥ 20	Major depression	GHQ ≥ 5, + those scoring GHQ 2–4 had a 30% probability & those scoring GHQ 0–1 had an 8% probability of selection. A random 50% of those not selected by GHQ but whom the GP had identified as having psychological problems were also selected	Non referred patients presenting with impairment due to anxiety or depression
**Measurement of depression at baseline**	SADS	-	Present State Exam (PSE)	SADS-Lifetime Version	Clinical Interview Schedule (CIS)	SCID, Clinical Global Impression (CGI), MADRS	Composite International Diagnostic Interview (CIDI), CES-D	CIDI, Somatic and Psychological Health Report (SPHERE)-34	SCID
**Cohort (% female)**	196 (% female not stated)	1336 [Data presented on 942 (74% female) insured patients with complete follow ups]	201 (64%–71% across onset groups) [Includes 20 participants with depression and 13 with borderline depression]	247 at first interview [Paper reports only on 166 followed up (54% female)]	305 (74%) [305 (74%) screened with GHQ in cross sectional study in 1997 (Kessler et al., 1999) [52% (157/305) GHQ +ve at screening, not clear from 2002 paper how many of the 157 were found again in 2002 paper] [60]]	492 (72%)	1117 (ranged across sites: 54–71%)	908 (66%)	108 (80%) [108 consecutive patients were prospectively evaluated, the paper does not state if this is the total number in cohort or those retained at follow up]
**Duration of follow-up**	12 months	6 months	3.5 years	6 months	3 years	6 months	12 months	12 months	8 months median follow up (Range 1–72 months)
**Other comorbidity measured**	No	Yes	Yes	Yes	Yes	Yes	Yes	Yes	Yes
**Care received examined**	No	Yes	Yes	Yes	Yes	Yes	Yes	Yes	Yes
**Definition of depression outcome**	Loss of key symptoms & syndromal status + return to normal functioning for a minimum of 2 months	Not stated	No longer met criteria for baseline diagnosis	Remitted cases were those with positive SADS-L diagnosis at baseline but not at follow up	No longer case on GHQ-12	Symptomatic exacerbation (MADRS score > 20) among patients who had responded to treatment but had not yet been well for a sufficient amount of time (under 6 months)	Complete remission from major depression	Results not yet available on outcome	Not stated
**Author**	**Michigan Depression Project [47, 49]**	**Parker et al. [68]**	**Ronalds et al. [37]**	**Rost et al. [67]**	**Rost et al. [38]**	**Schulberg et. [66]**	**Wagner et al. [42, 54]**	**WHO Collaborative Project on Psychological Problems in General Health Care [41,46,53,56,58,69,70]**	
**Location**	US	Australia	England	US	US	US	US	15 centres in 14 countries [Countries included in the study are: India, Turkey, Greece, Germany, The Netherlands [55], Nigeria, UK, Japan, France, Brazil, Chile, US [57,70], China & Italy [36]	
**Primary care setting/s**	Family physicians & University of Michigan, Department of Psychiatry Outpatient Depression Program	12 General Practices	One General Practice with an attached psychiatric social worker, a visiting psychiatrist & a clinical psychologist at the health centre	21 primary care practices	Using statewide telephone screening, identified and followed a cohort with a current major depression who made one or more visits to a primary care physician during the six months following baseline	One general medical clinic & two family practice clinics	One university-based family practice clinic	Health centre, primary health care unit, outpatient clinic, GP offices & private clinics, family practice, neighbourhood hospital & district hospital, primary care clinic	
**Selection of primary care setting**	Not stated	Not stated	Not stated	Not stated	Not stated	Not stated	Not stated	Previous successful WHO collaboration, research experience in primary care, access to patient population	
**Recruitment**	Waiting room	Consecutive patients	All surgery attenders	Consecutive patients	Statewide telephone screening, those who were depressed invited for telephone interview	Patients completed a depression screening instrument presented to them by receptionist	Patients introduced to RA by family physician at end of clinical visit	Consecutive patients	
**Screen for depression**	CES-D	Beck Depression Inventory (BDI)	GHQ-28	3-item screen for major depression & dysthymia	Burnam screener	CES-D	CES-D	GHQ-12	
**Exclusion criteria**	Not stated	Inadequate knowledge of English, severely distressed & first time attenders	Not meeting DSM-III-R criteria for generalised anxiety, panic or depressive disorder	No access to a telephone	Bereaved, manic, acutely suicidal or denied depressive symptoms	Contact with clinics during the six months prior to index assessment	Being seen by the Duke Student Health Service, employees of the Department of Community & Family Medicine, or too ill physically	< 18 years, > 65 years, too ill, no fixed address, did not come for a medical consultation, communication problem, no consent	
**Criteria for inclusion in cohort**	Major depression	BDI ≥ 10	Met DSM-III-R criteria for general anxiety, panic or depressive disorder	Major depression	Major depression	CES-D ≥ 16	CES-D ≥ 16 + random sample of CES-D <16	Current psychiatric disorder at baseline diagnostic assessment & 20% random sample	
**Measurement of depression at baseline**	SCID, CES-D, Hamilton Rating Scale for Depression (HAM-D^)^	past & current depression, PSE, Zung Depression Scale (ZDS) & 9 visual analogue scales	Psychiatric Assessment Schedule (PAS), Hamilton Depression Rating Scale (HDRS), Clinical Anxiety Scale (CAS)	Depression Outcome Module (DOS), Inventory to Diagnose Depression (IDD)	(Diagnostic Interview Schedule) DIS	DIS	DIS, CES-D	CIDI-Primary Health Care, GHQ-28	
**Cohort (% female)**	81 from primary care (% not stated)	35 (86%)	182 with depressive, anxiety or panic disorder [Reports on 148 (67% female) followed up]	47 (81%)	162 (% not stated)	294 (76%)	213 (range 61–83% across depression categories)	1174 (74%)	
**Duration of follow-up**	9 months	20 weeks	6 months	5 months	12 months	6 months	12 months	12 months	
**Other comorbidity measured**	Yes	Yes	Yes	Yes	Yes	Yes	Yes	Yes	
**Care received examined**	Yes	Yes	Yes	Yes	Yes	Yes	Yes	Yes	
**Definition of depression outcome**	Improvement in HAM-D score	improvement in Zung scores	Change in HDRS scores, changes in CAS scores & reduction in index of definition level	Remission from major depression	Remission: ≤ 2 of 9 DIS criteria for major depression met within last 2 weeks.	Resolution of major depressive disorder	Improvement i.e. moved to a less severe diagnostic category	Presence or absence of a depressive episode	

Studies varied widely in methods; including the screening and assessment instruments used, eligibility for inclusion in the cohort and the length of follow-up (Table 1). Cohort sizes ranged from 35 [68] to 1336 [51] patients. Follow-up ranged from 20 weeks [68] to 3.5 years [64]. The majority of the studies followed patients for 12 months, with nine studies following patients for less than 12 months (range 20 weeks to 9 months) [37,43,48,49,51,61,66-68] (Table 1).

### Setting

Most studies were undertaken in the US or Europe (Figure 2). The review also includes two multi-country studies: the Longitudinal Investigation of Depression Outcomes in primary care (LIDO) [44] and the World Health Organization (WHO) Collaborative Project on Psychological Problems in General Health Care [69]. Several of the study sites (Netherlands [55], Italy [36] and US [57,71]) involved in the multi countries WHO Collaborative Project on Psychological Problems in General Health Care have published results independently. The individual results are presented from these sites alongside the cumulative results from the WHO Collaborative Project on Psychological Problems in General Health Care in Table 2.

**Table 2:** Results

**Author**	**Blacker et al. [59]**	**Grembowski et al. [51]**	**Groningen Primary Care Study [32-35,50,64,65]**	**Kessler et al. [61]**	**Kessler et al. [45,60]**	**Limosin et al. [43]**	**Longitudinal investigation of depression outcomes in primary care (LIDO) [39,40, 44,52,62]**
**Retention**	72% (142/196) [Retention calculated from data presented in paper as not stated in results]	71% (942/1336)	77% (154/201)	67% (166/247)	59% (179/305) [Paper reports on 88 of original 157 GHQ +ve cases [60]]	86% (424/492)	87% (968/1117) [Data presented for 9 month follow up]
**Recovery from/improvement in depression**	% not stated	SCL-20 significantly reduced at 6 months (1.72 to 0.91, p < 0.001). Restricted activity days due to emotional health significantly reduced at 6 months (6.00 to 2.67 days, p < 0.001)	32% at 12 months & 47% at 3.5 years	At 6 months, major depression was present in 33% of both the new & continuing case groups & in 16% of the remitted group	50% (16/32) of those not detected by a GP at baseline or during the 3 year follow up	65% (308/476) recovered without relapse, 25% (117/476) developed a chronic condition & 11% (51/476) relapsed after recovery	35% (340/968) complete remission (range 25%–48% across sites) [Data presented for 9 month follow up]
**Predictors of outcome**	No data presented	No data presented	Positive life change increased the probability of remission in women fourfold (HR = 4.4), but not in men. Predictors of faster time to remission were low severity of pre-morbid difficulties (HR = 0.7), high self-esteem (HR = 1.4), and a coping style aimed at reducing tension (HR = 1.4) [32].	No data presented	No data presented	History of recurrent major depressive disorder was associated with a higher risk of relapse (OR 1.6, 95% CI 1.08–3.43)	Education, key life events, & the Quality of Life Depression Scale score at baseline predicted complete remission after adjusting for centres, socio-demographic data, severity of depression, comorbidity & general quality of life [52]
**Treatment**	61% received no treatment during follow up & 29% received treatments of a type intensity & degree that would be considered "therapeutic"	At follow up, 23% (219/942) of patients had been referred & 38% (356/942) had visited a mental health specialist. 54% visited a psychologist, 12% a psychiatrist & 34% visited both. Patients who saw a mental health specialist had more visits for depression to a primary physician than those who did not see a mental health specialist (1.93 [± SD 2.2] vs.0.98 [± SD 1.5]; p < 0.001)	Recognition of psychiatric disorder by a GP among new cases resulted in greater likelihood of referral to a mental health specialist (OR 3.0), receiving psychotropic medications (OR 4.5), having a counseling session (OR 12.2) and having any mental health treatment (OR 6.7) [64].	Patients developing disorders between the first & second interview had the highest total ambulatory use, primary & specialty care of all types (9.23) [5.95 visits for no diagnosis group, 7.53 visits for remitted cases & 8.35 visits for continuing cases], while patients with continuing cases had the highest mean number of primary care visits (4.54) [3.19 visits for no diagnosis group, 3.35 visits for remitted cases & 3.28 visits for new cases]	68% (38/56) with diagnosis, were treated with antidepressants & 21% (12/56) were referred to psychiatric services	All received an antidepressant treatment during the 6-month period. The total duration of treatment was <3 months for 41%, <30 days for 11%, between 30–60 days for 21%, & between 60–90 days for 9%. Duration of treatment was between 3–6 months for 14% of patients & ≥ 6 months for 45% of patients	0% (0%) in St Petersburg to 38% (33%) in Seattle received antidepressants (an effective dose) during follow up. 29% in Melbourne to 3% in St Petersburg received any specialty mental health care. The likelihood of receiving potentially effective antidepressant or mental health treatment at 3 months or 9 months did not differ across sites between the patients who were in complete remission & those who were not
**Author**	**Mental Health & General Practice Investigation (MaGPie) [63]**	**Manning et al. [48]**	**Michigan Depression Project [47, 49]**	**Parker et al. [68]**	**Ronalds et al. [37]**	**Rost et al. [67]**	**Rost et al. [38]**
**Retention**	83% (753/908), 77% final magpie interview (696/908)	108	73% (59/81) of primary care patients [Data presented for 4.5 month follow up]	57% (20/35)	81% (148/182), 74 with major depressive disorder and 74 with generalized anxiety or panic disorders	81% (38/47)	94% (152/162) [Paper reports on 98 patients visiting a primary care physician ≥ 1 during 6 months following baseline]
**Recovery from/improvement in depression**	No data presented	No data presented	Detected primary care patients failed to show significant improvement in HAM-D scores at 4.5 months. Both undetected primary care & detected psychiatric patients showed significant improvements over 4.5 months [By 9 months most patients in all 3 groups had improved & no longer met criteria for MDD [47]]	42.6 at baseline to 40.1 at follow up, indicating a 6% improvement	Median HDRS score reduced from 12 (interquartile range 9–15) at baseline to 5 five (interquartile range 1–10) at follow up	32% (12/38)	Remission: 36.9% of undetected patients and 29.2% of detected patients at 12 month follow up. Improvement: 10.2% of undetected patients and 9.8% of detected patients at 12 month follow up
**Predictors of outcome**	No data presented	No data presented	No data presented	Baseline predictors of a better outcome (improvement) were having a history of episodic or recurrent episodes; a more severe depression; lower social class; break up of an intimate relationship as a precipitant; a neutralizing life event & family support	A reduction in social difficulties, high baseline HDRS score, higher educational level and current employment were associated with greater reduction in HDRS scores (adjusted R2 = 33.2%).	Patients who received pharmacologic treatment concordant with guidelines between index visit & follow up were more likely to be in remission at follow up than subjects who did not (X^2 ^= 3.8, p < 0.05)	No data presented
**Treatment**	In the year preceding the index consultation, 1/3 of male & 42% of female patients had five or more GP consultations	Not purpose of paper	In the past 6 months: 75% of detected & 9% of non-detected primary care patients had been prescribed medication, 88% & 29% respectively had been counseled by physician, 56% & 12% had been referred for counseling & 36% & 3% respectively had received individual or group therapy	At follow up, 2 were still receiving antidepressant & 4 anxiolytic medication, while 3 were continuing to see their GP for depression	In 93 patients the psychiatric disorder was recognised & managed as follows: 30 patients by discussion/counseling without drugs, 26 treated by GP with psychotropic drugs & 37 patients were referred to the specialist services. The greatest reduction in depression was in the patients managed without psychotropic drugs & referred to mental health services.	63% (24/38) filled prescriptions for one or more antidepressant medications between index visit & follow up. 29% (11/38) received pharmacologic treatment concordant with AHCPR guidelines. Two patients received 3 or more counseling sessions from a mental health professional.	52% of detected patients had a prescription for antidepressant medication during the year following baseline, 27% completed course in accordance with guidelines. 7% of detected patients received a referral from their primary care physician to a mental health specialist in addition to receiving a prescription for antidepressant medication.
**Author**	**Schulberg et. [66]**	**Wagner et al. [42, 54]**	**WHO Collaborative Project on Psychological Problems in General Health Care [41,46, 53,56,58,69,70]**	**WHO Collaborative Project on Psychological Problems in General Health Care (Italy) [36]**	**WHO Collaborative Project on Psychological Problems in General Health Care (US) [57,71]**	**WHO Collaborative Project on Psychological Problems in General Health Care (Netherlands) [55]**	
**Retention**	93% (274/294)	86% (184//213)	62% (729/1174) of patients with depressive episode				
**Recovery from/improvement in depression**	71% (12/17)	37% (19/51) of patients with major depression at baseline were asymptomatic at 12 months & 56% (37/66) of patients with minor depression at baseline were asymptomatic at 12 months [42]	67% (482/725)	Of the 29 participants with baseline threshold major depression, 21% (n = 7) were well at 12 months & 14% (n = 4) were subthreshold	At the 12-month assessment, 15/50 (30%) patients continued to satisfy criteria for major depressive disorder, 4/50 (8%) met criteria for minor depression, & 31/50 (62%) did not satisfy criteria for any depressive disorder	At 12 months, 32% (21/66) of patients with a GP recognized ICD-10 diagnosis recovered (50% (33/66) improved) & 27% (21/79) patients whose ICD-10 diagnosis was not recognized by a GP recovered (47% (37/79) improved)	
**Predictors of outcome**	Psychiatric status at initial assessment & the number of assigned medical diagnoses rather than the physicians recognition & treatment of depression strongly predict continued affective disorder	Risk for persistent depression at 12 months for those with major depression at baseline was 44% greater in those with co-existing anxiety disorder (RR 1.44, 95% CI 1.02–2.04 [42]	Less than 6 years formal education, unemployment, severity of depression, antidepressant use, repeated suicidal thoughts & abdominal pain as main reason for contacting GP were related to depression at follow up [41]. Comorbid generalised anxiety had a negative influence on the long-term course of depression in men (OR 2.66) but not women (OR = 0.52) [58]. At three month follow up, recognized patients reported a significantly greater reduction in GHQ scores than unrecognized patients (6.1 vs 4.1, F = 5.33, df = 1, p = 0.02), however by 12 month follow up there was no difference between recognized and unrecognized patients in change in GHQ score or change in diagnostic status from baseline. Patients with unrecognized and/or untreated depression showed rates of improvement similar to those of patients with recognized and/or treated depression [46].	Recognition of mental disorder by the physician at baseline was not associated with an improvement of psychopathology after 12 months, but was associated with an improvement in occupational disability & self-reported disability among threshold cases	The likelihood of complete remission (no depressive diagnosis at 12-month follow-up) was 60% (21 of 35) for the recognized group & 68% (10 of 15)	Patients whose psychological disorder was recognized had no better outcomes than those whose disorder was not recognized	
**Treatment**	Among the 9 of 13 patients (depression not recognized by GP) whose depression remitted, 5 received no antidepressants, one received only 25 mg of Imipramine, 2 received an anti-anxiety drug & one received sleeping medications. 3 received psychiatric treatment at a psychiatric facility during the study period	Odds for a visit to a mental health specialist or in the general medical sector for mental health purposes were significantly higher for respondents with a diagnosis of major depression relative to respondents with minor depression & significantly lower again for asymptomatic respondents, again relative to the minor depression group. In multivariate modeling, female gender, white race, & higher education was associated with higher odds of a mental health visit [54]	Those depressed at follow-up were twice as likely to be taking antidepressants (20%) as non-depressed at follow- up (11%)	Not presented	Of patients with major depressive disorder (n = 64), pharmacy records showed that a total of 36 (56%) received antidepressant medications at some time during the 3 months following screening. Of them, 28 (78%) received dosages within the recommended ranges & 27 (75%) continued to refill antidepressant prescriptions for at least 90 days. Both dosage & duration of treatment met these standards in 22 (61%) of 36 cases. Among those with major depression, 39% (23/59) of visited at least one specialty mental health since screening. 66% (39/59) received some treatment during the 3 month follow up. Likelihood of receiving treatment was strongly related to severity of illness.	Not presented	

### Selection procedures

Consecutive and convenience sampling methods were used in the majority of studies. No study recruited a random sample of patients from primary care. Only six studies detailed how the settings or clinicians were selected: GPs were randomly selected in two studies [43,63]; settings were selected in two studies based on research experience and capacity, and on previous collaboration [44,69]; GPs were a representative sample from the total population of GPs in the area in one study [64] and in another, GPs were a consenting sub-sample from a larger study on physician referral [51]. Those remaining were conducted on convenience samples selected from health centres [59]; general practices or family practice clinics [37,45,48,54,66,68], with rural practices [67], from family physicians and a University Psychiatry Outpatient Department [49] or in multi-specialty clinics that had mental health care services, outpatient services, day care and inpatient services [61]. Using statewide telephone screening, one study identified and followed a cohort with a current major depression who made one or more visits to a primary care physician during the six months following baseline [38].

### Inclusion criteria

Six studies included only patients with major depression [38,43,44,49,59,67], three studies included patients with depressive symptoms [51,66,68], two studies included patients with depression or anxiety disorders or symptoms [37,48] and one included 'new' (i.e. a psychiatric diagnosis had not been diagnosed during the 12 months prior to the index visit) and 'old' (i.e. a psychiatric diagnosis had been diagnosed during the 12 months prior to the index visit) patients with depression and anxiety disorders, including borderline disorders and non-specific psychiatric symptoms [35]. Of the remaining five studies, four included patients with depressive symptoms and asymptomatic patients in the follow-up [45,54,61,63] and one included patients with current psychiatric disorder and a random sample of patients without a current psychiatric disorder in the follow-up [69]. Two studies excluded patients who had received recent treatment for depression; one in the previous three months [44] and one where patients had contact with the clinic where the research was being conducted in the six months prior to the study [66]. One study included only non-referred patients presenting with anxiety or depression [48]. One study screened people for depression from random households using state-wide telephone lists and presented follow-up data on those who had visited a general practitioner in the six months following baseline interview [38].

### Screening procedures

Seventeen different instruments were used at baseline to measure depressive symptoms or disorders. The Center for Epidemiologic Studies-Depression Scale (CES-D) [72] and various versions of the General Health Questionnaire (GHQ) [73] were the most commonly used screening instruments (Table 1).

### Comorbidity measures

The majority of studies also measured co-morbid psychiatric symptoms or disorders, mainly anxiety related. Only six examined physical co-morbidities or days out of role [38,44,48,54,67,69]. Grembowski et al. [51] reported measuring 21 co-morbid conditions, however these conditions were not reported.

### Treatment and health service use

Only one study did not report on the care received by patients [59]. Two of the sixteen studies that report collecting data on health service use did not report the findings [38,61]. While Kessler *et al*. [61] reviewed medical records, their purpose was to determine point recognition and validate mental disorders given in the context of an associated physical disorder, not to examine care received. Rost *et al*. [38] examined medical, pharmaceutical and insurance records to determine the detected and undetected depression during follow-up. Five studies examined medication use [44,48,63,68,69] and two presented data on the use of antidepressant medication [51,67]. Seven studies examined health care/service utilization [44,49,54,63,66,67,69] and one described use of and referral to mental health specialists [51]. Parker *et al*. [68] examined GP and psychiatric care, and four studies examined GP treatment [37,45,63,64]. The MaGPie Research Group [63] also examined barriers to care and patients' attitudes to their GP. Seven studies asked patients to self report on the care they received between baseline and follow-up [37,44,51,54,63,66,69], four studies asked the primary care physician to report on care [43,49,63,64] and eight reviewed medical records, chart evidence, insurance records and/or pharmaceutical records [37,38,45,48,51,63,66,67] [results not mutually exclusive]. Parker *et al*. [68] collected data on medication, GP and psychiatric care during follow-up, however they did not report whether the data were patient self-report, physician report or a review of records.

### Methodological quality of the studies included in the review

Many of the studies have methodological limitations, including small sample sizes (range 35–108 patients at baseline and 20–59 patients at follow-up) [48,49,67,68] and small numbers in the cohort with depression [35,66]. Furthermore, Schulberg *et al*. [66] was the only study to consider characteristics of the sample screened with the primary care population to determine whether the study patients were representative. No study randomly selected general practices and then approached a random selection of their patient list to avoid the frequency of attendance bias present in studies recruiting consecutive patients. Previous research has highlighted that a high proportion of eligible patients are missed when recruiting patients from general practice waiting rooms, thus limiting the generalisability of the findings [74]. Moreover, only three studies included a random or representative sample of primary care physicians [43,63,64], and seven studies recruited patients from just one centre or general practice [37,45,48,54,59,61,66]. These methodological limitations must be acknowledged when considering whether findings from the studies included in this review can be generalised to primary care populations.

### Representativeness of samples to the primary care population

Only one study was able to compare characteristics of the sample screened with the primary care population [66]. Schulberg *et al*. [66] compared their cohort of patients with depressive symptoms with the total clinic population and noted the cohort was younger and had more females than would be expected from the medical facilities where the research took place. Rost *et al*. [38] compared patients who agreed to take part in the baseline interview with those patients who were eligible but refused, and reported no differences in socio-demographic data and clinical characteristics including the severity of depression, except that the cohort were younger and more likely to live in metropolitan areas.

Between 62%–91% of patients were retained in the studies at 12 months, and 67%–93% at six months (Table 1). However, as some cohorts included asymptomatic patients [45,54,61,63,69], the power of some studies to determine predictors of depression outcomes is limited.

### Course of depression

#### History and duration

Limosin *et al*. [43] reported that the current episode of depression was not the first for 38% of the patients in their study. In that study, the average number of previous depressive episodes was 2.1 (SD 1.7 episodes; range 1–12) and the average time reported between the first and current depressive episode was 5.9 years (SD 5.8 years; range 0.5 to 30 years).

Only four of the 17 studies provide information on the chronicity of depressive symptoms [37,43,58,65]. The mean duration of the current episode of depression varied across the two studies that reported it. Limosin *et al*. [43] reported the average length of the current episode of depression was 2.8 months (SD 7 months; range 0.5 to 8 years), while in the Groningen Primary Care Study, the mean episode duration for depressive disorders was 9 to 10 months [65], however the small sample size in the Groningen study and non-random selection of patients in both studies limits the generalisability of these findings. Ronalds *et al*. [37] reported a greater improvement in depressive disorder for patients with a depressive disorder of less than six months duration compared to patients with a duration of greater than six months. In the Groningen Primary Care Study, patients with depression, anxiety or neurasthenia disorders with a recent onset or exacerbation, were twice as likely to have that disorder recognized by a GP and to have improved at follow-up, than patients with chronic psychological disorders [64].

#### Recovery

Eight studies have presented data on recovery from major depressive disorder. Among these, 32% of patients had recovered at four months [67], 65–71% had recovered at six months [43,61,66], 35% had recovered at nine months [52], 32%–67% had recovered at 12 months [41,42,54,64,69,70] and 47% had recovered at 3.5 years [35] (Table 2). It is not possible to meaningfully compare the findings across studies as there was no consistency in methods, with studies using different instruments for screening and diagnosis, and different methods of recruitment and administration (clinician administered, researcher administered or self report) of instruments (Table 1). Furthermore, four of these studies included small numbers of patients with depression; Schulberg et al. [66] followed up 17 patients with major depressive disorder, Ormel et al. [35] followed up 20 patients with depression and 13 with borderline depression, Rost et al. [67] followed up 38 patients with major depressive disorder and Wagner et al. [54] followed up 51 patients with major depression and 66 with minor depression. There were three studies with large sample sizes that presented data on recovery from major depression/depressive disorder. In France, Limosin et al. [43] found that 65% (308/476) of patients with major depressive disorder recovered without relapse at six months; at nine month follow-up, the LIDO study [52] conducted in six countries, found that 35% (340/968) of patients reported complete remission from major depressive disorder (ranged from 25%–48% across countries); and in the WHO Collaborative Project on Psychological Problems in General Health Care conducted in 14 countries, 67% (482/725) of patients with depressive episodes recovered at 12 months [41]. Although both the LIDO and WHO studies administered (different versions of) the CIDI, the results of the studies are very different. The authors of the LIDO study offer no explanation for the lower rate of recovery in their study compared to other studies [52], however these follow-ups were done at nine and 12 months respectively which may contribute to the recovery rates.

The interpretation of the findings on recovery is further complicated when recovery rates are compared for patients whose depression was detected or undetected [38,46,49,64,66]. Despite the methodological limitations of some of the studies presented such as small sample size; the results suggest there is no difference in depression outcomes between patients whose depression is recognized or unrecognized. Rost et al. [38] reported that 47.2% of undetected patients and 39% of detected patients no longer met criteria for major depression at 12 month follow-up. At six month follow-up, Schulberg [66] found that *"psychiatric status at initial assessment and the number of assigned medical diagnoses rather than the physician's recognition and treatment of depression strongly predict continued affective disorder" *(p.312), however, only 6.2% and 2.9% of this cohort had major depressive disorder at baseline and follow-up respectively. In the WHO Psychological Problems in General Health Care study conducted in 14 countries, Simon et al. [46] found that at baseline recognized patients had significantly higher mean GHQ scores and were more disabled than unrecognized patients. At three month follow-up, recognized patients reported a significantly greater reduction in GHQ scores than unrecognized patients; however by 12 month follow-up there was no difference between recognized and unrecognized patients in change in GHQ score or change in diagnostic status from baseline. The authors conclude that *"recognition and appropriate diagnosis of depression in primary care is associated with significantly greater short-term improvement [and] that increasing recognition of depression in primary care is only a first step toward more appropriate treatment" *(p.97). In the Groningen Primary Care Study at 12 month follow-up psychopathology had improved for 75% of patients whose psychological disorder was recognized (n = 100) by a GP compared to 33% of unrecognized patients (n = 79) (p < 0.001) (OR 6.1 for PSE-ID) [64]. A similar pattern was found with improvement in social disability: a significantly greater proportion of recognized patients compared to unrecognized patients reported improvement at 12 months (56% vs. 24%, p < 0.001) (OR = 4.0). The majority of participants in this study were 'new' cases (i.e. a psychiatric diagnosis had not been diagnosed during the 12 months prior to the index visit) which may explain in part why the results conflict with the results from the other studies presented.

#### Relapse rates

Only two studies in the current review presented data on relapse rates [32,43]. Limosin *et al*. [43] reported at six months that 65% (308/476) of patients with major depressive disorder had recovered without relapse, 25% (117/476) developed a chronic condition and 11% (51/476) relapsed after recovery. In the Groningen Primary Care Study, 93% of depressed patients had remitted from index episode at 12 months [32] and the relapse (described as *"transition from an asymptomatic state of at least two months to a state of mental disorder"*) rate among depressed patients was 30%, however the cohort included only 20 participants with major depression. Limosin *et al*. [43] found that a history of recurrent major depressive disorder was associated with a higher risk of relapse at six months, while Parker *et al*. [68] found patients with episodic or recurrent episodes were more likely to improve at 20 weeks than those with other patterns of depression, however due to this study's small sample size and short follow-up time (20 weeks) the results should be considered tentatively.

### Risk factors for the course of major depressive disorder and depressive symptoms

Six studies examined the predictors of the course of depression [32,41-43,52,58,67].

#### Chronicity of depression

Longer pre-baseline duration of the depressive episode in the WHO Collaborative Project on Psychological Problems in General Health Care study was a predictor of a poor course of depression [58]. Multivariate analysis reported that among those whose pre-baseline duration was at least one year compared to those whose pre-baseline duration was less than three months, the odds of a poor short-term course of depression (no full recovery within half a year) were over five times higher (versus those whose pre-baseline duration was less than three months) (OR = 5.22, 95% CI 2.45–11.15). The same was found for long-term outcomes, with those who had a pre-baseline duration of one year being more likely to report a poor outcome compared to those whose pre-baseline duration was less than one year (OR = 3.54, 95% CI 1.67–7.52). In the Groningen Primary Care study, duration of index episode was not associated with the occurrence of a relapse within the 12 month follow-up after remission [32].

#### Severity of depression

Wagner et al. [54] found that a greater proportion of patients with minor depression (56%, 37/66) than major depression (37%, 19/51) at baseline were asymptomatic at 12 months. They found that a diagnosis of minor depression was associated with almost the same degree of impairment in health status, functional status and disability, and psychiatric service utilization as a diagnosis of major depression. However, 20% (13/66) of patients with minor depression at baseline met criteria for major depression at 12 months, while 22% (11/51) of patients with major depression at baseline met criteria for minor depression at 12 months. The authors conclude that sub-threshold depression or the persistence of depressive symptoms is a risk for developing major depression. In the Groningen Primary Care Study, 31% of patients with borderline depression had recovered at 12 months and 70% at 3.5 years [35]. Indeed, the Groningen Primary Care Study [35] found that partial remission rather than complete recovery *"was the rule and was associated with residual disability" *(p.759). This study also found that depression had better outcomes than anxiety and mixed anxiety-depression. At baseline, patients with both anxiety and depression reported the highest symptoms levels on the Present State Exam. However given the small sample size with each disorder these results should be interpreted with caution.

The data from studies measuring depressive symptoms are also difficult to compare for similar reasons. Parker et al. [68] reported a 6% improvement in depressive symptoms at 20 weeks; and others reported that depressive symptoms had significantly reduced at six month follow-up [37,51]. At four and a half month follow-up, one study found a significant reduction in depressive symptom scores among primary care patients whose depression was not detected compared to no significant reduction in depressive symptom scores among detected patients [49]. Kessler et al [45] reported that of the 88 patients who met criteria for a case on the GHQ, 50% (16/32) of those not detected by a GP at baseline or during the three year follow up, were no longer cases at three year follow-up. Grembowski et al. [51] and Ronalds et al. [37] retained a large sample of patients at follow-up, however Grembowski et al. [51] only included insured patients and therefore their findings are limited to a sample of mainly middle-class, Caucasian adults with depressive symptoms. The other three studies followed up small numbers of patients and may not have had sufficient power to determine reduction in depressive symptoms [45,49,68].

There were two studies where improvement in depressive symptoms was presented [37,68]. Parker et al. [68] found that baseline predictors of a *"better outcome" *for the 20 patients with depressive symptoms at baseline who were followed up for 20 weeks were: having a history of episodic or recurrent episodes; a more severe depression; lower social class; break up of an intimate relationship as a precipitant; a neutralizing life event and family support. Multivariate analysis conducted by Ronalds et al. [37] found that at six month follow-up, high baseline depression score, higher educational level and current employment were associated with greater reduction in depression score among patients with major depressive disorder and generalized anxiety or panic disorders at baseline. The factors associated with outcome in this study were not reported for patients with each disorder, therefore it is difficult to draw any firm conclusions about the factors associated with improvement in depression among depressed patients.

#### Comorbidity

Gaynes et al. [42] reported that the risk for persistent depression at 12 months for those with major depression at baseline was 44% greater in those with co-existing anxiety disorder (RR = 1.44, 95% CI 1.02–2.04). In the Groningen Primary Care Study, half of the patients who experienced a positive life change remitted within four months [32], the probability of remission was 2.3 times higher following positive life change (HR = 2.3). The positive life change increased the probability of remission in women fourfold but not in men (HR = 4.4). Multivariate analysis found that quicker time to remission was associated with low severity of pre-morbid difficulties (HR = 0.7), high self-esteem (HR = 1.4), and a coping style aimed at reducing tension (HR = 1.4).

#### Treatment

Rost et al. [67] reported that patients with major depression who received pharmacologic treatment concordant with guidelines between baseline and five month follow-up were more likely to be in remission at follow-up than subjects who did not, however the sample size was small and of the 38 patients followed up, only 11 received such treatment. The findings on whether being prescribed antidepressants was associated with recovery were conflicting. Rost *et al*. [67] reported that patients who received pharmacologic treatment concordant with guidelines between index visit and five month follow-up were more likely to be in remission at follow-up than subjects who did not, while Barkow *et al*. [41] found antidepressant use was related to persisting depression at 12 month follow-up. The WHO Mental Disorders in General Health Care Study found that while patients receiving antidepressants reported significantly less symptoms on the GHQ at three months than patients receiving sedatives, this was not the case at 12 months [56]. However the authors highlight that as the study was not a trial, efficacy of psychoactive drugs cannot be inferred.

#### Sex

Despite the majority of patients in the 17 studies being female, only three studies reported on outcome by sex [37,41,68]. All three studies reported no difference between depression outcome for males and females at follow-up. However two of these studies may not have had sufficient power to detect differences between males and females [37,68].

#### Predictors of the course of depression from multivariate analyses

Whilst there are difficulties in comparing results across the three large scale studies that measured risk factors for persistence or recovery from depression [41,43,52,58], some conclusions can be drawn. Remission from depression at nine months was associated with higher level of education (OR = 1.06, 95% CI 1.051–1.11), higher quality of life (OR = 0.94, 95% CI 0.92–0.97) and experiencing key life events (OR = 0.71, 95% CI 0.66–0.83) in the LIDO study, after adjusting for centres, socio-demographic data, severity of depression, co-morbidity and general quality of life [52]. A significantly greater proportion of patients whose major depression had remitted at nine month follow-up had medical conditions, dysthymia or anxiety disorders than patients who were not in complete remission. While the authors found that there was no consistent variable that predicted remission across the six country sites, they believed this may have been a result of the *"modest" *sample size (range in cohort sizes by country 142–185). In the WHO Collaborative Project on Psychological Problems in General Health Care study, sustained non-remission (i.e. presence of a non-remitted or new depression) at 12 month follow-up was associated with lower levels of education (0 years versus 11+ years: OR = 3.78, 95% CI 1.83–7.79; 1–5 years versus 11+ years OR = 1.81, 95% CI 1.02–3.19), unemployment (employed versus unemployed: OR = 1.57, 95% CI 1.02–2.43), severity of depression (severe versus moderate: OR = 3.27, 95% CI 1.91–5.62), antidepressant use (OR = 1.79, 95% CI 1.06–3.03), repeated suicidal thoughts ("crossed my mind" versus no suicidal thoughts) (OR = 1.82, 95% CI 1.14–2.93), and abdominal pain as main reason for consulting the general practitioner (OR = 2.30, 95% CI 1.17–4.52) [41]. The study also reported that patients had a greater probability of a poor long term course (no recovery over the 12 month follow-up period) if the severity of their depression was moderate or worse (versus mild) (OR = 3.38, 95% CI 1.49–7.65), their pre-baseline duration was greater than one year (versus less than one year) (OR = 3.54, 95% CI 1.67–7.52), they did not have a chronic physical illness (OR = 0.31, 95% CI 0.13–0.73), they had low social support (versus high/average) (OR = 0.4 5, 95% CI 0.19–1.07), and they had lower levels of education (≥ 13 years versus < 10 years) (OR = 0.18, 95% CI 0.07–0.47) [58]. A previous episode of depression increased the probability of chronicity for younger (OR = 3.60, 95% CI 0.92–14.14) but not older (OR = 0.28, 95% CI 0.05–1.45) patients. They found that among patients with co-morbid anxiety, depressed women had a smaller probability of chronicity than depressed men (OR = 0.13, 95% CI 0.04–0.41) [58]. Limosin et al reported that relapse from depression was associated with a history of recurrent major depressive disorder at six month follow-up (OR = 1.6, 95% CI 1.08–3.43) [43].

Risk factors for persistence of depression identified in this review were: severity and chronicity of the depressive episode, the presence of suicidal thoughts, antidepressant use, poorer self-reported quality of life, lower self-reported social support, experiencing key life events, lower education level and unemployment.

### Treatment and health service use

The proportion of patients receiving antidepressant medication during the study follow-up period ranged from 0% (St Petersbourg site in Fleck *et al*. [52]) to 100% [43] (Table 2). However, the proportion of these patients prescribed antidepressants according to guidelines in the three studies that reported this, ranged from 27% [38] to 61% [41].

Three studies reported that the likelihood of receiving treatment was associated with severity of illness [51,54,71]. In addition, The WHO Mental Disorders in General Health Care Study found that younger age, being male and less time since first onset were associated with not being prescribed psychoactive drugs [56]. Grembowski et al. [51] found that more severe depressive symptoms at baseline, previously attending a mental health specialist, more years of education, younger age and being female were the best predictors of referral and utilization of a mental health specialist and that managed care was not associated with a reduced likelihood of referral to or of visiting a mental health specialist. Another study found major depression (OR 1.83), female gender (OR 2.17), white race (OR 2.34), and higher education (OR 1.21) were associated with higher odds of a mental health visit in the last four months [54]. The US site of the WHO Collaborative Project on Psychological Problems in General Health Care found that participants with higher symptom severity as measured by the GHQ-28 at baseline, and more disability, were more likely to receive antidepressant medication or use any specialty mental health services [71]. This study also reported that patients with anxiety or depressive disorders at baseline had higher health care costs in the six months prior to baseline (US $2,390) than patients with sub-threshold (US $1,098) or no disorders (US $1,397). These cost differences were due to higher use of general medical services rather than higher mental health treatment costs [57]. In the Groningen Primary Care Study, recognition of psychiatric disorder by a GP among new cases resulted in greater likelihood of referral to a mental health specialist (OR 3.0), receiving psychotropic medications (OR 4.5), having a counseling session (OR 12.2) and having any mental health treatment (OR 6.7) [64].

## Discussion

Understanding the complex interplay between the development and persistence of depression over the longer term, psychological, social and physical factors and the health service use and treatment patterns is crucial if we are to plan better models of care to cope with the increasing burden that depression and related disorders is placing on people experiencing the condition, their social networks and the health care system.

Despite the growing interest in depression being managed as a chronic illness in primary care; this review identified only 17 observational studies of depression in primary care, most of which have been conducted in Europe or the US. The striking finding of this review is the small sample size of many studies, the small numbers in the cohort with depression and the short length of follow-up. The studies provide information on the nature and course of depression for around 7,500 people receiving routine primary care. Of the 17 studies, nine studies followed almost 3,000 patients from four countries for less than 12 months, six followed almost 4,000 patients from 19 countries for 12 months, and two followed over 500 patients from two countries for longer than 12 months. Only five studies included large sample sizes (greater than 400 patients) [43,44,51,63,69] and only three of them reported risk factors for the course of depression [41,43,52,58].

The review aimed to identify risk factors for persistence of depression that were common across studies. This was difficult as the factors studied and measurement tools used varied widely. Few studies included psychiatric, physical and social risk factors together, thus preventing us from reporting on the relative importance of each of these. Based on this review, of the factors studied, it appears that the severity and chronicity of the depressive episode, the presence of suicidal thoughts, antidepressant use, comorbid physical illness, poorer self-reported quality of life, lower self-reported social support, negative life events, lower education level and unemployment are all factors associated with the persistence of depression.

Several gaps in the studies included in this review have been identified. In particular there is inconsistency in the way depression is defined (symptoms or disorders), how it is measured and the risk factors that are studied. Non-psychiatric co-morbidities, social and contextual factors have been poorly explored. Health service use and treatment is not well documented and studies lack patients' qualitative experience of depression.

The 17 studies can be grouped into two major types; those that focus on the nature and course of depressive symptoms and those that focus on the nature and course of major depressive disorder. Recent research by Simon *et al*. [19] suggests that the more prevalent conditions, such as minor depression and dysthymia, place a greater burden on the health care system than the less prevalent major depression; yet, many studies reviewed focused on individuals experiencing major depression. The debate regarding use of diagnostic categories versus symptom severity in research and clinical practice is ongoing [75,76] and studies that include the capacity to measure both will add valuable information to assist researchers and clinicians as we develop future classificatory systems and clinical guidelines. We urgently need better consistency in the terminology used in reported research as even among studies including patients with major depression in the cohort, the terminology used varied across cohorts (Table 1).

The review found that for some, depression is a chronic and relapsing disorder, with studies reporting recovery from a major depressive disorder at 12 months for between one to two thirds of patients. The variation in recovery across studies may be due in part to the different methods used in each study, or because people recruited into each cohort differ. As none of the studies recruited a random sample of patients, the generalisability of findings is problematic.

The studies reviewed highlight the complex and changing nature of depression as it exists in a primary care sample; symptoms improve and deteriorate over time (how this relates to treatments received is difficult to judge) and patients can oscillate between depression categories.

We are unable to reliably report on relapse rates asonly two studies report relevant data stating rates of 11% at six monthsand 30% at 12 months. Establishing reliable estimates ofdepression relapse in the primary care setting requires follow-up of larger samplesover a longer time frame.

It is widely reported that women experience depression about twice as much as men [77], despite this none of the studies reviewed reported on risk factors for persistence of depression for males and females separately. Given the higher prevalence of depression among females, studies should analyse the results by sex whenever the sample size allows.

Current guidelines for management of depression in primary care are constructed for use, in the main, with newly diagnosed cases of depression that are not complicated by physical comorbidities and social factors. This review demonstrates that newly diagnosed cases of depression are relatively uncommon, that physical comorbidities are common and that social factors, when studied are associated with poorer outcomes. Future guidelines should take into account the findings of the naturalistic studies and not rely solely upon evidence gathered in randomized controlled treatment trials.

## Conclusion

Naturalistic studies that document the personal experience, treatment and service use and take account of the psychological, physical and social factors influencing depression outcomes are essential for future service planning. We hope this review will assist others to plan their studies and enable them to address the methodological limitations of previous research.

## Competing interests

The author(s) declare that they have no competing interests.

## Authors' contributions

GG and JG agreed the search terms for the review, GG searched the electronic databases and articles were assessed as relevant by both authors. GG and JG drafted the manuscript. Both authors read and approved the final manuscript.

## Pre-publication history

The pre-publication history for this paper can be accessed here:


